# Poor prognostic factors for relapse of interstitial lung disease with anti-aminoacyl-tRNA synthetase antibodies after combination therapy

**DOI:** 10.3389/fimmu.2024.1407633

**Published:** 2024-09-13

**Authors:** Shogo Matsuda, Takuya Kotani, Katsumasa Oe, Ayana Okazaki, Takao Kiboshi, Takayasu Suzuka, Yumiko Wada, Takeshi Shoda, Tohru Takeuchi

**Affiliations:** Department of Internal Medicine IV, Division of Rheumatology, Osaka Medical and Pharmaceutical University, Osaka, Japan

**Keywords:** interstitial lung disease, forced vital capacity, aldolase, poor prognostic factors, chest CT

## Abstract

**Introduction:**

This study aimed to identify useful clinical indicators for predicting the relapse of interstitial lung disease (ILD) complicated with anti-aminoacyl-tRNA synthetase (ARS) antibodies (anti-ARS-ILD), being treated with prednisolone and calcineurin inhibitors.

**Methods:**

Fifty patients with anti-ARS-ILD were enrolled between October 2014 and August 2022. All patients were treated with prednisolone and calcineurin inhibitors as remission induction therapy and followed up for over a year with these combination therapies. We examined patients who experienced ILD relapse after immunosuppressive treatment. We explored the risk factors for predicting ILD relapse in these patients by comparing demographic, clinical, laboratory, and radiological findings and treatments between the relapsed and non-relapsed groups on admission.

**Results:**

Of the 50 patients, 19 (38%) relapsed during a median follow-up of 4.8 years. Univariate and multivariate Cox regression analyses identified the presence of acute/subacute (A/S)-ILD, higher serum aldolase (ALD) and surfactant protein-D (SP-D) levels, and lower %forced vital capacity (FVC) as risk factors for relapse in patients with anti-ARS-ILD. Using the receiver operating curve analysis, ALD ≥6.3 U/L, SP-D ≥207 ng/mL, and %FVC ≤76.8% were determined as the cut-off levels for indicating a poor prognosis. The 5-year relapse rate was significantly higher in patients with A/S-ILD, serum ALD≥6.3 U/L, serum SP-D ≥207 ng/mL, or %FVC of ≤76.8% than in those without these parameters. (*P*=0.009, 0.0005, 0.0007, 0.0004, respectively) Serum ALD levels were significantly correlated with the disease activity indicators of anti-ARS-ILD.

**Conclusion:**

The presence of A/S-ILD, higher serum ALD and SP-D levels, and lower %FVC are useful indicators for predicting anti-ARS-ILD relapse.

## Introduction

Polymyositis (PM) and dermatomyositis (DM) are idiopathic inflammatory myopathies (IIM) of various tissues, including the skin, heart, and lung (1, 2). Clinically amyopathic dermatomyositis (CADM) is a subgroup of DM characterized by a typical skin rash, such as heliotrope rash and Gottron’s sign, with few or no clinical symptoms of myositis (3, 4). Interstitial lung disease (ILD) is a life-threatening condition that frequently accompanies PM/DM/CADM (5). PM and DM/CADM are recognized as common types of inflammatory myopathies, and inflammatory myopathy-specific autoantibodies are recently used to classify subtypes of patients with IIM (6, 7).

Autoantibodies against aminoacyl-tRNA synthetase (ARSs), including anti-PL-7, PL-12, anti-OJ, and anti-EJ, anti-KS, anti-Zo, and anti-Ha, are a subset of myositis-specific autoantibodies (8). The clinical manifestations at onset have been reported to differ based on the type of anti-ARS autoantibody (9). The presence of anti-ARS autoantibody is used as a criterion for the diagnosis of the antisynthetase syndrome (ASS), which is characterized by the occurrence of various organ involvements, such as inflammatory myositis, ILD, arthritis, and mechanic’s hands (10, 11). ASS is considered to be classified separately from PM and DM due to its specific clinical manifestations, as only about 20% of patients with ASS have muscle involvement, which is often complicated by ILD lesions (9, 12). ILD with a positive anti-ARS antibody (anti-ARS-ILD) typically responds well to immunosuppressive therapy, and has a good response in a short-term but a high relapse rate in a long-term (13, 14). Previous reports showed that high serum Krebs von den Lungen-6 (KL-6) levels, serial KL-6 increases, and the presence of middle lobe traction bronchiectasis on high-resolution computed tomography (HRCT) were associated with the deterioration of myositis-associated ILD, including anti-ARS-ILD (15, 16). However, biomarkers for predicting the relapse of anti-ARS-ILD have not been fully elucidated.

In the treatment of anti-ARS-ILD, prednisolone (PSL) monotherapy has been associated with relapse (17). Calcineurin inhibitor (CNI), including cyclosporin-A (CSA) and tacrolimus (TAC), is reported to be effective for treatments of patients with anti-ARS-ILD with corticosteroid-refractory ILD, and combination therapy with PSL and a CNI is recommended as maintenance therapy in anti-ARS-ILD (18–20). Response to combination therapy is heterogeneous, with some patients relapsing after receiving the combination therapy (16, 21). Little is known about the risk factors for relapse in patients with anti-ARS-ILD following combination therapy.

This study examined relapsed patients undergoing combination therapy with PSL and CNI inhibitors and compared the clinical characteristics on admission between the relapsed and non-relapsed groups. Herein, we showed that the prevalence of acute/subacute (A/S)-ILD and the high serum aldolase (ALD) and surfactant protein-D (SP-D) levels and lower %FVC were useful indicators for predicting relapse in anti-ARS-ILD after combination therapy.

## Materials and methods

### Patients

We examined patients admitted to Osaka Medical and Pharmaceutical University Hospital between October 2014 and August 2022. The diagnosis of PM, ADM, or CADM was made based on the Bohan and Peter criteria (1, 2) or criteria outlined by Sontheimer and Gerami et al. (3, 4) The diagnosis of interstitial pneumonia with autoimmune-features (IPAF) was made using the criteria of the ERS/ATS task force (22). ACR/EULAR classification criteria were used to check whether they were classified as “definite IIM” or “probable IIM” (6). Patients with malignancies were excluded from this study. The presence of ILD was assessed using HRCT. ILD can be divided into acute/subacute ILD (A/S-ILD) and chronic ILD (C-ILD). A/S-ILD was defined as rapid worsening of the respiratory condition, laboratory pulmonary function tests, arterial blood gas findings, and chest HRCT images within 3 months of onset (23). On the contrary, C-ILD does not fulfil the definition of A/S-ILD. Disease duration was defined as the period between the onset of symptoms associated with anti-ARS-ILD and hospital admission. Clinical data were obtained from the medical records. This study was conducted in accordance with the Declaration of Helsinki and its amendments and it was approved by the Ethics Committee of Osaka Medical and Pharmaceutical University (approval no. 1529). Informed consent was obtained from each patient.

### Arterial blood gas analysis and pulmonary function testing

Arterial blood gas analysis, including PaO2, PaCO2, and alveolar-arterial oxygen difference (AaDO2), was performed at admission. Respiratory function, including forced vital capacity (FVC) and diffusion capacity of the lungs for carbon monoxide (DLco), was measured using spirometry (SYSTEM21; Minato Medical Science, Osaka, Japan), as previously described (24). Respiratory function test results are expressed as percentages of the predicted value.

### HRCT scoring and HRCT pattern

HRCT was performed using a 64-detector row CT Aquilon multiscanner (Toshiba Medical Systems Corporation, Tokyo, Japan). The slice thickness was 1.0–1.5 mm every 10 mm, and the scan area included the entire lung. All patients underwent chest HRCT before treatment, and the images were reviewed independently by three observers (TK, TSu, and TSh) who were blinded to the patients’ clinical information. Inter-observer disagreements were resolved by consensus. Ground-glass opacity (GGO) and fibrosis were scored to assess the HRCT findings as previously described (25). Each patient’s lobe was scored by the same observer, and the average value was used. The scores were then summed to obtain the total CT score.

Nonspecific interstitial pneumonia (NSIP) and organizing pneumonia (OP) are typical characteristics in anti-ARS-ILD, so we classified the HRCT pattern into NSIP, OP, and NSIP with OP pattern, as previously reported (16, 26). All patients underwent chest HRCT prior to treatment, and images were reviewed independently by 2 pulmonologists (TK, and TS) blinded to the patients’ clinical information.

### Measurement of laboratory parameters

The levels of creatine kinase (CK), ALD, lactic acid dehydrogenase (LDH), C-reactive protein (CRP), KL-6, SP-D, and ferritin were measured. Anti-ARS antibodies were examined using ELISA (MESACUP; MBL, Nagoya, Japan) and a blot assay (Myositis Profile Euroline Blot Test Kit; EUROIMMUN, Lübeck, Germany). Autoantibodies (antigen including Jo-1, PL-12, PL-7, EJ, Ro-52) were measured by this immunoblotting.

### Treatments

PSL (0.5–1.0 mg/kg/day) was administered to all the patients. CSA or TAC were used as a combination therapy at the physician’s discretion. CSA was started at 4 mg/kg/day, once daily, before breakfast, and the concentration 2 h after administration was adjusted to 1,000 ng/mL or higher. TAC was started at 0.1 mg/kg/day twice a day before breakfast and dinner, and the trough concentration was adjusted to 5–10 ng/mL. Additional treatments, such as methylprednisolone (MPDN) pulse therapy, intravenous cyclophosphamide pulse therapy (IVCY), intravenous immunoglobulin (IVIG), plasma exchange (PE), and mycophenolate mofetil (MMF), were administered based on each patient’s condition at the physician’s discretion.

### Definitions of relapse and responders/non responders

PSL was progressively tapered according to the clinical effectiveness after remission induction therapy. In all cases, clinical and biochemical evaluation was performed every 1-2 months. Relapse of ILD was defined when both of the following conditions were fulfilled after the remission induction therapy: the appearance of a new GGO on chest HRCT and the requirement of intensified treatment (15, 27, 28).. We excluded other conditions which could cause ground glass opacities on HRCT, including cardiac failure, fluid overload, and pulmonary embolism (27). A concrete example of a relapse in chest HRCT is shown in **Supplementary Figure 1**.

Moreover, we defined “responders” as patients who responded to remission induction therapy without disease exacerbation and were discharged, and defined “non responders” as those who did not respond to remission induction therapy with exacerbation of interstitial shadows on HRCT (29).

### Statistical analysis

The data are presented as the median (interquartile range). Fisher’s exact test was used when appropriate, and the Mann–Whitney U test was used to compare median values. We used receiver operating characteristic (ROC) curve analysis to determine the most suitable cut-off level for predicting anti-ARS-ILD relapse. We used the Kaplan–Meier method to assess survival curves and the log-rank test to evaluate the significance of differences between the two groups. The correlations were evaluated using Spearman’s correlation coefficients. A P-value of <0.05 was considered significant. Data were analysed using JMP (version 17.0; SAS Institute Inc., Cary, NC, USA) and GraphPad Prism (version 8.0; GraphPad Software, La Jolla, CA, USA).

## Results

### Clinical characteristics of patients with anti-ARS-ILD

**Figure 1:**
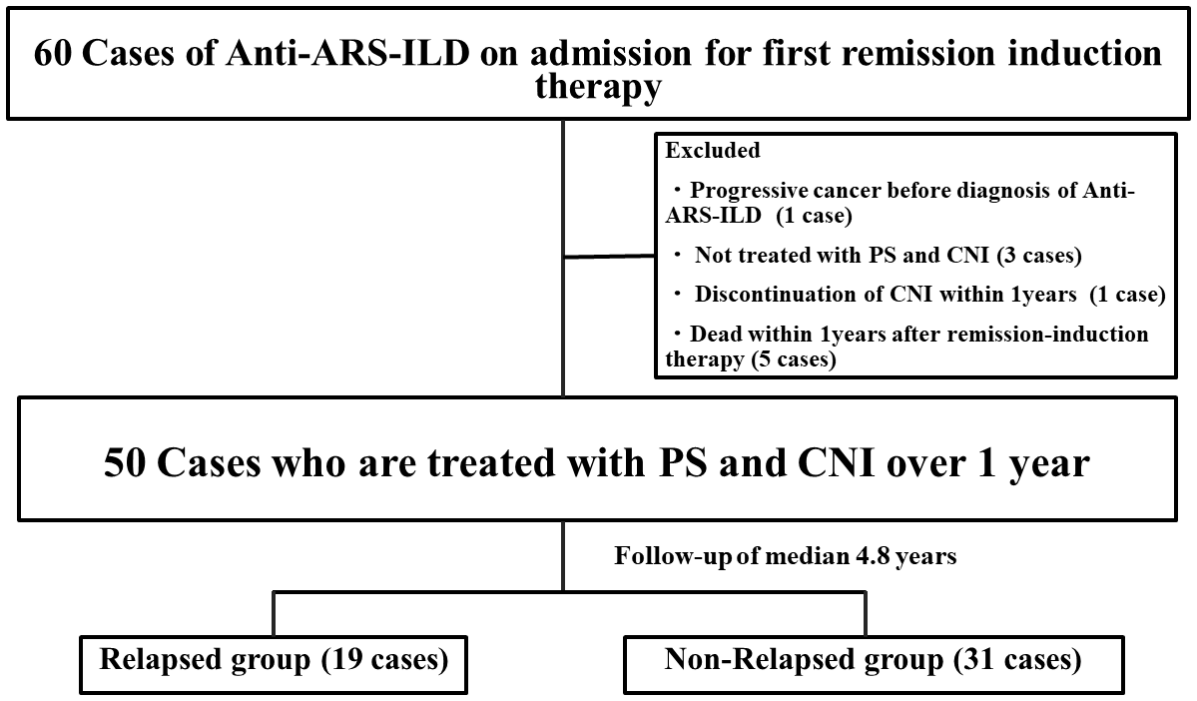
Flowchart of this study. Flowchart illustrating the selection and the prognosis of patients with anti-ARS-ILD. ARS, Aminoacyl-tRNA Synthetase; ILD, interstitial lung disease; PS, prednisolone; CNI, calcineurin inhibitor.

A flowchart illustrating the selection and prognosis of patients with anti-ARS-ILD is shown in **Figure 1**. Sixty patients with anti-ARS-ILD were admitted to our hospital for the first remission induction therapy between October 2014 and August 2022, and 10 patients were excluded for the following reasons: coexistence of progressive cancer before the diagnosis of anti-ARS-ILD (1 case), not being treated with PSL and CNI (3 cases), discontinuation of IS within 1 year (1 case), and death within 1 year after remission induction therapy (5 cases). The clinical characteristics of 50 patients with anti-ARS-ILD are shown in **Table 1**. The median patient age was 61 years, and 70% of the patients were women. Eighteen percent of patients were “definite IM” and 44% patients were “probable IIM” using ACR/EULAR criteria (6). Sixty percent (30 patients) had A/S-ILD, and 40% (20 patients) had C-ILD. All patients with C-ILD underwent therapeutic intervention because of the gradual progression of ILD from more than 3 months to several years. The median disease duration was 3.2 months. The median serum levels of various parameters were as follows: CK, 100 U/L (reference ranges: 41–153 U/L); ALD, 6.3 U/L (reference ranges: 2.1–6.1 U/L); CRP, 0.29 mg/dL (reference ranges <0.14 mg/dL); KL-6, 1,022 U/mL (reference ranges: 105–401 U/mL); SP-D, 167 ng/mL (reference ranges: <110 ng/mL); ferritin, 132 ng/mL (reference ranges: 6.2–138 ng/mL). In the PFT findings, the median percentage predicted for FVC and DLco was 81.6% and 51.2%, respectively. The HRCT pattern included NSIP in 54%, NSIP with OP overlap pattern in 42%, and OP in 4%. There were no patients who had classified as usual interstitial pneumonia pattern.

**Table 1:** Clinical characteristics with anti-ARS-ILD at baseline.

Characteristics	anti-ARS-ILD (n= 50)
Age, years	61(51-67)
Female, n (%)	35(70)
CADM/DM/PM/IPAF, n (%)	23 (46.0)/5 (10.0)/1 (2.0)/21 (42.0)
A/SILD, n (%)	30 (60.0)
Antibody (Jo-1/EJ/PL7/PL12/others)	13/16/12/3
Antibody (Ro52)	32 (64.0)
Disease duration, months	3.2 (1.5-4.8)
Modified MRC scale	1 (0-3)
Laboratory findings	
CK, U/L	100(61-224)
ALD, U/L	6.3(3.8-12.5)^a^
CRP, mg/mL	0.29(0.1-1.9)
KL-6, U/mL	1022 (564-1755)
SP-D, ng/mL	167 (111-277)^b^
Ferritin, ng/mL	132 (79-344)^c^
AaDO2	19.0 (6.6-28.7) ^d^
PFT findings	
Percent predicted FVC, %	81.6 (65.9-91.1)^e^
Percent predicted DLco, %	51.2 (44-64.9)^f^
HRCT pattern, n (%)	
NSIP	27 (54.0)
NSIP with OP overlap	21 (42.0)
OP	2 (4.0)
HRCT score	
Total GGO score	5.8 (4.3-9.5)
Total Fibrosis score	2 (1.3-3.8)

The laboratory markers are presented as the median (interquartile range). ARS, Aminoacyl-tRNA Synthetase; ILD, interstitial lung disease; CADM, clinically amyopathic dermatomyositis; DM, dermatomyositis; PM, polymyositis; IPAF, interstitial pneumonia with autoimmune-features; MRC, Medical Research Council; CK, creatine kinase; ALD, aldolase; CRP, C-reactive protein; KL-6, Krebs von den Lungen-6; SP-D, Surfactant Protein-D; A-aDO2, alveolar-arterial oxygen difference; FVC, forced vital capacity; DLCO, diffusion capacity of the lung for carbon monoxide; NSIP, nonspecific interstitial pneumonia; OP, organizing pneumonia; GGO, ground-glass opacity. ^a^Number of subjects, n= 48. ^b^Number of subjects, n= 39. ^c^Number of subjects, n= 46. ^d^Number of subjects, n= 48. ^e^Number of subjects, n= 41. ^f^Number of subjects, n= 39.

### Comparison of clinical characteristics and contents of treatment between relapsed group and non-relapsed group

All patients were treated with prednisolone and calcineurin inhibitors as remission induction therapy and followed up for over a year with these combination therapies. (**Figure 1**) All patients were responders in this study. During a median follow-up of 4.8 years, 19 patients relapsed after remission induction therapy. The median time from initiation of remission induction therapy to relapse of ILD was 2.5 years. The median dose of prednisolone was 6 mg/day, and that of CNI, including TAC and CSA, was 2.5 mg/day and 150 mg/day, respectively. We compared the clinical and laboratory findings between the relapsed (19 patients) and non-relapsed (31 patients) anti-ARS-ILD groups (**Table 2**). No significant differences were observed in age, sex, or disease duration. The proportion of patients with A/S-ILD was higher in the relapsed group (79.0%) than that in the non-relapsed group (48.4%; *P*=0.04). Sixty-two percent of patients with anti-Jo-1 antibody, 56% of patients with anti-EJ antibody and 60% of patients with anti-PL-7/PL-12 antibodies were A/S-ILDs. There were no significant differences in the ratio of A/S-ILD for the type of anti-ARS antibody. Additionally, the initial serum levels of ALD and SP-D were higher in the relapsed group (8.6 U/L, 248 ng/mL, respectively) than those in the non-relapsed group (5.0 IU/L, 147 ng/mL) (*P*=0.004, 0.009, respectively). There were no significant differences in the initial serum levels of CK, CRP, KL-6, ferritin, or AaDO2 between the two groups. There were no significant differences in the ratio of the type of anti-ARS antibody between the two groups. Also, there were no significant differences in the anti-Ro52 positivity between the two groups. In the pulmonary function test results, the %FVC was significantly lower in the relapsed group (70%) than that in the non-relapsed group (84.9%; *P*=0.02) There were no significant differences in the total GGO and fibrosis score between the relapsed and non-relapsed groups. There were no significances in HRCT pattern between them.

**Table 2:** Comparison of clinical characteristics, and outcomes of patients between relapsed group and non-relapsed group in anti-ARS-ILD.

Characteristics	Relapsed group (n= 19)	Non-Relapsed group (n=31)	*P* value
Age, years	56(45-67)	63(54-68)	0.15
Female, n (%)	12(63)	23(74)	0.53
CADM/DM/PM/IPAF, n (%)	7(36.8)/2 (10.5)/1 (5.3)/9 (47.4)	16(51.6)/3(9.7)/0(0)/12 (38.7)	0.49
A/SILD, n (%)	15 (79.0)	15 (48.4)	0.04*
Antibody(Jo-1/EJ/PL7/PL12/others)	6/5/4/1	7/11/8/2	0.85
Antibody (Ro52)	13 (68.4)	19 (61.3)	0.76
Disease duration, months	2.9 (1.7-4.2)	3.3 (1.0-5.1)	0.94
Modified MRC scale	2(1-3)	1 (0-2)	0.19
Laboratory findings			
CK, U/L	132 (65-343)	85 (56-132)	0.08
ALD, U/L	8.6(6.3-24.8)^a^	5.0(3.7-8.5)^b^	0.004**
CRP, mg/mL	0.6(0.1-1.5)	0.1 (0.05-2.5)	0.44
KL-6, U/mL	1376 (772-2480)	924 (495-1399)	0.06
SP-D, ng/mL	248 (197-455) ^c^	147 (108-195) ^d^	0.009**
Ferritin, ng/mL	151(85-499)^e^	124 (48-286) ^f^	0.28
AaDO2	21.1 (15.6-38.5)^g^	13.6 (4.6-26.7)^h^	0.13
PFT findings			
Percent predicted FVC, %	70 (61.0-84.1) ^i^	84.9 (78.3-94.1)^j^	0.02*
Percent predicted DLco, %	45.7 (39.2-51.5)^k^	54.9 (45.3-66.9) ^l^	0.05
HRCT pattern, n (%)			
NSIP	9 (47.4)	18 (58.1)	0.75
NSIP with OP overlap	9 (47.4)	12 (38.7)	
OP	1 (5.3)	1 (3.2)	
HRCT score			
Total GGO score	6 (4.3-10.7)	5.7 (4.0-9.3)	0.65
Total Fibrosis score	2 (1.7-3.3)	2 (1-4)	0.94

The laboratory markers are presented as the median (interquartile range). The P-values were estimated using Fisher’s exact test or Wilcoxon rank sum test. *P < 0.05, **P < 0.01. ARS, Aminoacyl-tRNA Synthetase; ILD, interstitial lung disease; CADM, clinically amyopathic dermatomyositis; DM, dermatomyositis; PM, polymyositis; IPAF, interstitial pneumonia with autoimmune-features; MRC, Medical Research Council; CK, creatine kinase; ALD, aldolase; CRP, C-reactive protein; KL-6, Krebs von den Lungen-6; SP-D, Surfactant Protein-D; A-aDO2, alveolar-arterial oxygen difference; FVC, forced vital capacity; DLCO, diffusion capacity of the lung for carbon monoxide; NSIP, nonspecific interstitial pneumonia; OP, organizing pneumonia; GGO, ground-glass opacity. ^a^Number of subjects, n= 18. ^b^Number of subjects, n= 30. ^c^Number of subjects, n= 14. ^d^Number of subjects, n= 25. ^e^Number of subjects, n= 17. ^f^Number of subjects, n= 29. ^g^Number of subjects, n= 19. ^h^Number of subjects, n= 29. ^i^Number of subjects, n= 16. ^j^Number of subjects, n= 25. ^k^Number of subjects, n= 14. ^l^Number of subjects, n= 25.

We compared the contents of treatment between relapsed group and non-relapsed group (**Supplementary Table 1**). The median initial PSL dose was significantly higher in the relapse group than in the non-relapse group. (*P*=0.02). Moreover, there were no differences observed in the doses of CSA or TAC or the frequency of MPDN, IVCY, PE, or MMF administration between the two groups.

### Cox regression analysis of relapse in anti-ARS-ILD

A/S-ILD, higher serum ALD and SP-D levels, and lower %FVC were identified as risk factors in the univariate analysis. Next, we performed a Cox regression analysis using these risk factors. Univariate analysis using a Cox regression model showed that A/S-ILD, higher serum ALD and SP-D levels, and lower %FVC were predictors of ILD relapse in patients with anti-ARS-ILD (*P*=0.01, 0.0007, 0.01, and 0.0007, respectively; **Table 3**).

**Table 3:** Cox regression analysis of relapse in anti-ARS ILD.

	Unadjusted			Age, Disease duration-Adjusted		
Risk Factors	Hazard ratio	95% CI	*P*	Hazards ratio	95% CI	*P*
A/S ILD	3.97	1.31-12.1	0.01*	14.45	3.11-67.10	0.0007***
ALD, U/L (per 1 unit increase)	1.02	1.01-1.03	0.0007***	1.02	1.01-1.03	0.0004***
SP-D, ng/mL (per 1 unit increase)	1.002	1.0003 -1.004	0.01*	1.003	1.0004-1.005	0.01*
%FVC (per 1 unit increase)	0.93	0.89-0.97	0.0007***	0.92	0.88-0.96	0.0005***

The Hazard ratios of relapse were derived from univariate and multivariate analysis with a cox regression model. Covariates: age, disease duration. *P < 0.05, **P < 0.01, ***P < 0.001. ARS, Aminoacyl-tRNA Synthetase; ILD, interstitial lung disease; ALD, aldolase; SP-D, Surfactant Protein-D; FVC, forced vital capacity.

Previous reports have shown that ageing and disease duration from disease onset to therapeutic intervention are associated with poor prognosis in patients with PM/DM-ILD (30, 31). We performed multivariate analyses using Cox regression analysis to determine whether A/S-ILD, higher serum ALD and SP-D levels, and lower %FVC were independently associated with ILD relapse after adjusting for age and disease duration. After adjusting for these covariates, multivariate analysis using a Cox regression model also revealed that A/S-ILD, higher serum ALD and SP-D levels, and lower %FVC were independent risk factors for relapse in patients with anti-ARS-ILD (*P*=0.0007, 0.0004, 0.01, and 0.0005, respectively; **Table 3**).

### Cut-off values for aldolase, %FVC and survival rates

To estimate the cut-off points for assessing factors related to the poor prognosis of anti-ARS-ILD, ROC curve analysis was performed using ALD, SP-D, and %FVC. The level that maximised the area under the ROC curve was 6.3 U/L for ALD (area under the curve [AUC]: 0.75, sensitivity: 83.3%, specificity: 66.7%), 207 ng/mL for SP-D (AUC: 0.75, sensitivity: 78.6%, specificity: 80%), and 76.8% for %FVC (AUC: 0.72, sensitivity: 62.5%, specificity: 80%). Thus, ALD ≥6.3 U/L, SP-D ≥207 ng/mL, and %FVC ≤76.8% were the best cut-off levels for indicating a poor prognosis. The ROC curves of ALD, SP-D, and %FVC for differentiating between the relapsed and non-relapsed groups are shown in **Supplementary Figure 2**.

**Figure 2:**
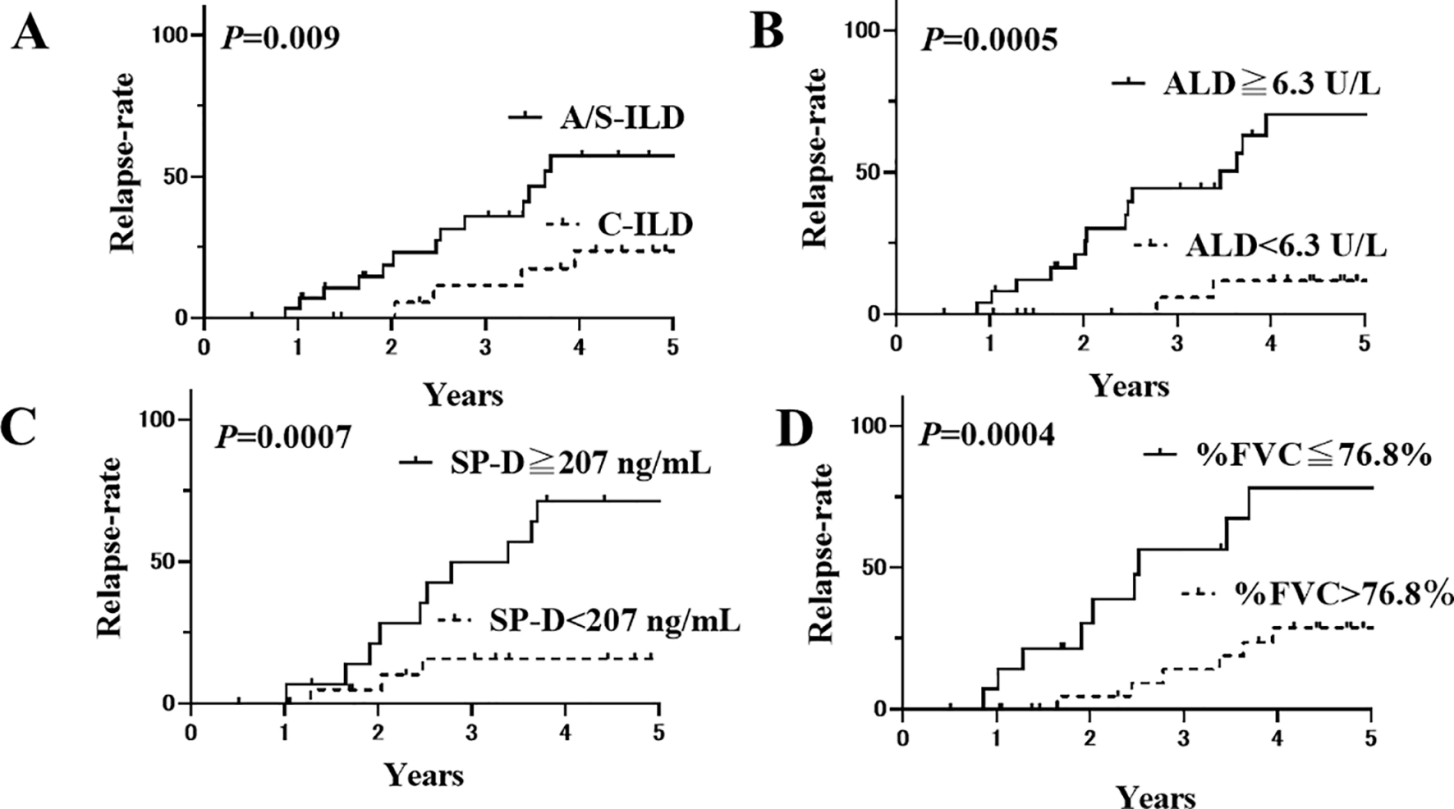
Kaplan-Meier curves of anti-ARS-ILD patients based on their initial clinical characteristics. **(A)** The relapse rate after 5 years for patients with A/S-ILD (57.3%) was significantly higher than that for patients with C-ILD (23.7%) (*P*=0.009). Solid line: with A/S-ILD, dashed line: with C-ILD. **(B)** The relapse rate after 5 years for patients with an initial ALD ≥ 6.3 U/L (70.3%) was significantly higher than that for patients with ALD < 6.3 U/L (11.8%) (*P*=0.0005). Solid line: ≥ 6.3 U/L, dashed line: < 6.3 U/L. **(C)** The relapse rate after 5 years for patients with an initial SP-D ≥ 207 ng/mL (71.3%) was significantly higher than that for patients with SP-D < 207 ng/mL (15.7%) (*P*=0.0007). Solid line: ≥ 207 ng/mL, dashed line: < 207 ng/mL. **(D)** The relapse rate after 5 years for patients with an initial %FVC of ≤ 76.8% (78.2%) was also significantly higher than that for patients with %FVC of ≻76.8% (28.7%) (*P*=0.0004). Solid line: %FVC of ≤ 76.8%, dashed line: %FVC of ≻76.8%. ARS, Aminoacyl-tRNA Synthetase; ILD, interstitial lung disease; A/S, acute/subacute; C, chronic; ALD, aldolase; SP-D, Surfactant Protein-D; FVC, forced vital capacity.

  The patients were then divided into two groups based on these cut-off levels, and Kaplan–Meier survival curves were plotted (**Figures 2A–D**). The 5-year relapse rate was significantly higher in patients with A/S-ILD (57.3%) than in those with C-ILD (23.7%; *P*=0.009) The 5-year relapse rate was significantly higher in patients with ALD ≥6.3 U/L (70.3%) than in those with ALD <6.3 U/L (11.8%; *P*=0.0005; **Figure 2B**). The 5-year relapse rate was significantly higher in patients with SP-D ≥207 ng/mL (71.3%) than in those with SP-D <207 ng/mL (15.7%; *P*=0.0007; **Figure 2C**). The 5-year relapse rate was significantly higher in patients with %FVC of ≤76.8% (78.2%) than in those with %FVC of ≻76.8% (28.7%). (*P*=0.0004; **Figure 2D**).

### Correlation between serum aldolase levels and disease severity indicators of anti-ARS positive ILD

A previous report showed that patients with ASS often present with fasciitis-dominant myopathy with elevated serum ALD levels, and myofascitis was more likely to be observed in patients with DM with rapid progressive ILD (RP-ILD) than in those without RP-ILD (32, 33). Based on these findings, we examined whether serum ALD levels were correlated with disease severity indicators, including CRP, ferritin, KL-6, SP-D, %FVC, %DLco, and HRCT scores, in patients with anti-ARS-ILD on admission, as shown in **Table 4** (24). Serum ALD levels were significantly and positively correlated with CRP (R=0.51), ferritin (R=0.49), and total GGO scores (R=0.34; *P*=0.0002, 0.0005, and 0.02, respectively). Moreover, they were significantly negatively correlated with %FVC (R=-0.46) and %DLco (R=-0.41; *P*=0.003 and 0.009, respectively). In contrast, there were no significant correlations between serum CK levels and these biomarkers.

**Table 4:** Correlation between serum myopathy marker and disease activity of anti-ARS positive-ILD.

	CRP	ferritin	KL-6	Sp-D	%FVC	%Dlco	Total GGO	Total Fib
Aldolase	0.51***	0.49***	0.09	–0.1	–0.46**	–0.41**	0.34*	–0.02
CK	0.08	0.2	–0.11	–0.26	–0.22	–0.24	–0.03	–0.1

Statistical analyses were performed using Spearman’s rank correlation coefficient. *P < 0.05, **P < 0.01, ***P < 0.001. ARS, Aminoacyl-tRNA Synthetase; ILD, interstitial lung disease; CRP, C-reactive protein; KL-6, Krebs von den Lungen-6; SP-D, Surfactant Protein-D; FVC, forced vital capacity; DLCO, diffusion capacity of the lung for carbon monoxide; GGO, ground-glass opacity; Fib, Fibrosis.

## Discussion

This study showed that 38% of the patients with anti-ARS-ILD relapsed during follow-up. The prevalence of A/S-ILD, serum ALD levels, and SP-D levels were significantly higher, and the %FVC on admission was significantly lower in the relapsed group than in the non-relapsed group. Multivariate analyses revealed that A/S-ILD, higher serum ALD levels, and lower %FVC were independently associated with anti-ARS-ILD relapse. Serum ALD levels were significantly correlated with disease activity indicators, including CRP, ferritin, %FVC, %DLco, and GGO scores on HRCT.

Fujisawa et al. reported that A/S-ILD is a poor prognostic factor in PM/DM/CADM-ILD (34). The prevalence of A/S-ILD varies from 30–50% in previous studies (16, 35, 36), and the association of A/S-ILD with the risk of relapse after treatment has not been elucidated in patients with anti-ARS-ILD. The present study showed that A/S-ILD at disease onset was also a high-risk factor for relapse after remission therapy in patients with anti-ARS-ILD.

Chronic onset is more common in patients with anti-ARS-ILD; however, some patients also present with acute onset (37, 38). Previous reports showed that ILD with anti-Jo-1 antibody, anti-PL7/PL12 antibody, or anti-EJ antibody often presented with acute onset, leading to poor prognosis (39–41). Tillie-Leblond et al. reported that 47% of patients with ILD and anti-Jo-1 antibodies presented with acute onset and were likely to progress to ILD 12 months after treatment (39). Marie et al. also showed that the extent of fibrosis on chest HRCT in the anti-PL7/PL12 positive group was more severe, and acute onset and deterioration were more frequent than in the anti-Jo-1 antibody-positive group (40). Sasano et al. reported that 75% of patients with anti-EJ antibody-related ILD presented with acute onset, and 50% relapsed after immunosuppressive therapy (41). These studies are consistent with those of this study. In our study, anti-Jo-1 antibody, anti-PL7/PL12 antibody, or anti-EJ antibody were positive in almost 90% of the patients, and nearly 40% of the patients with anti-ARS-ILD relapsed despite combination therapy with PSL and CNI. These results suggest that patients with anti-ARS-ILD who develop A/S-ILD may require more intensive remission induction therapy and a slow reduction of immunosuppressive drugs during maintenance therapy.

High serum ALD levels indicate severe muscle and myofascial inflammation in patients with DM (33). Fukamatsu et al. reported that patients with DM with anti-ARS antibodies had a higher rate of ILD complications and a higher percentage of elevated serum ALD levels and were more likely to have fasciitis-dominant myopathy compared to those without anti-ARS antibodies (32). Karino et al. suggested that myofasciitis and RP-ILD may be caused by microvasculopathy of the lungs and muscles in patients with DM because patients with RP-ILD were more likely to have myofascitis than those without RP-ILD (33). The present study revealed that serum ALD levels were significantly higher in the A/S-ILD group than in the C-ILD group and were significantly correlated with disease severity indicators of PM/DM-ILD. Serum ALD levels can be useful biomarkers for predicting ILD relapse and assessing the severity of myositis.

SP-D is secreted by alveolar type II epithelial cells, and serum SP-D levels increase owing to alveolar-vascular leakage in patients with pulmonary fibrosis (42, 43). Serum SP-D levels reflect the disease activity and were indicators of relapse of ILD (43, 44). Two previous reports showed that high serum levels of SP-D were associated with a poor prognosis in PM/DM-ILD (45, 46).. Ihn et al. previously reported that increases in serum SP-D were accompanied by ILD exacerbations in 75% of patients with PM/DM-ILD (45). Arai et al. showed that an increase in serum SP-D levels during the first 4 weeks after treatment was a poor prognostic factor for PM/DM-ILD (46). Our study also indicated that high serum SP-D levels on admission were associated with a higher relapse rate after remission induction therapy for anti-ARS-ILD. However, the serum KL-6 level on admission was not a prognostic indicator of relapse in this study. Serum SP-D levels peaked within the first 4 weeks after immunosuppressive therapy, whereas KL-6 levels increased for up to 3 months after treatment in patients with PM/DM-ILD (46). Therefore, serum SP-D levels may be a more sensitive predictor of relapse than serum KL-6 levels in anti-ARS-ILD. However, further investigation is required to confirm this hypothesis.

The association between %VC and the relapse of myositis-associated ILD has been reported in several studies. Takanashi et al. reported that myositis-associated ILD patients with %VC <70.5% had a higher relapse rate than those with %VC ≧70.5% (15). Nakazawa et al. reported that the %VC at baseline was significantly lower in the early recurrence group than in the non-early recurrence group (17). Marie et al. reported that patients with ILD deterioration had lower %FVC values than those without PM/DM-ILD (47). Our study supports these previous studies by showing that %FVC is a useful predictor of anti-ARS-ILD deterioration.

Anti-Ro52 antibodies positivity is significantly associated with the presence of ILD in patients with ASS, but the clinical relevance of anti-Ro52 antibodies has not been elucidated (48). In this study, we evaluated the association between relapse rate and the anti-Ro52 antibodies positivity, but there were no association between them. There are contrasting results regarding the relationship between anti-Ro52 antibodies positivity and prognosis in ASS, so further studies are needed to prove this (49, 50).

NSIP and OP pattern are frequently observed on HRCT in patients with ASS, as our study also showed. Debray, et al. reported that 38% patients with ASS-ILD progressed fibrosis even after the decrease or disappearance of consolidation on HRCT (21). Therefore, progressive fibrosing ILD (PF-ILD) is a serious clinical phenotype in anti-ARS-ILD, which is determined by rapid deterioration of respiratory symptoms, lung function and progressive fibrosis on HRCT (51). Several reports showed that 10-20% of IIM-ILD, including ASS, met the criteria of PF-ILD or progressive pulmonary fibrosis (52, 53). Patients with PF-ILD have a high-mortality rate, but risk of PPF progression in IIM-ILD has not been elucidated. These predictive indicators in our study may be applicable for progressive fibrosing ILD with anti-ARS antibodies, and further investigations are needed to identify this.

Our study has a few limitations. First, all patients were Japanese, and the study number was limited. Therefore, it may result in selection bias, and it is unclear whether these findings would correspond to other ethnicities. Second, these data might have been affected by indication bias because treatment strategies for ARS-ILD were determined based on the physician’s discretion. Third, these data might be affected by tertiary care bias because 60% of patients with anti-ARS-ILD had A/S-ILD. Fourth, the classification criteria for ASS are varied according to studies (54). In our study, anti-ARS-ILD patients were included in this study, so we did not include the ASS patients without ILD but with arthritis. This may cause the selection bias in the study of ASS. It is crucial to investigate whether these three indicators are useful for predicting relapse in patients with anti-ARS-ILD in a multi-centre study. Finally, we used the appearance of a new GGO on chest HRCT as a criterion for ILD relapse, as previously described (15, 28). However, the definition of ILD relapse on chest HRCT findings varies in studies; thus, our definition of relapse may have affected the results (17, 19, 55). Despite these limitations, the present study highlights useful indicators of anti-ARS-ILD relapse.

## Conclusions

We revealed that the presence of A/S-ILD, higher serum ALD and SP-D levels, and lower %FVC were independently associated with anti-ARS-ILD relapse. Further investigations are needed to determine the useful indicators for predicting the relapse of ILD in anti-ARS-ILD patients in a large, prospective, multi-centre study.

## Data Availability

The raw data supporting the conclusions of this article will be made available by the authors, without undue reservation.

## Supplementary material

Supplementary Figure 1 The representative images of relapse in patients with anti-ARS-ILD. **(A)**, chest HRCT image on admission. **(B)**, chest HRCT image after remission induction therapy. **(C)**, chest HRCT image on relapse. On chest HRCT, GGO was seen in bilateral lower lobes (wide arrow). ARS, Aminoacyl-tRNA Synthetase; ILD, interstitial lung disease; HRCT, high-resolution computed tomography; GGO, Ground-glass opacity.

Supplementary Figure 2 The ROC curves of aldolase **(A)**, SP-D **(B)**, %FVC **(C)** for differentiating patients with relapse from patients without relapse in anti-ARS-ILD. ROC, receiver operating characteristic; SP-D, surfactant protein-D; FVC, forced vital capacity; ARS, Aminoacyl-tRNA Synthetase; ILD, interstitial lung disease; AUC, area under the curve.
