# Supplementary figures and images for: Poor prognostic factors for relapse of interstitial lung disease with anti-aminoacyl-tRNA synthetase antibodies after combination therapy
Source: Front Immunol. 2024 Sep 13;15:1407633. doi: 10.3389/fimmu.2024.1407633 (PMC11427292; doi:10.3389/fimmu.2024.1407633)

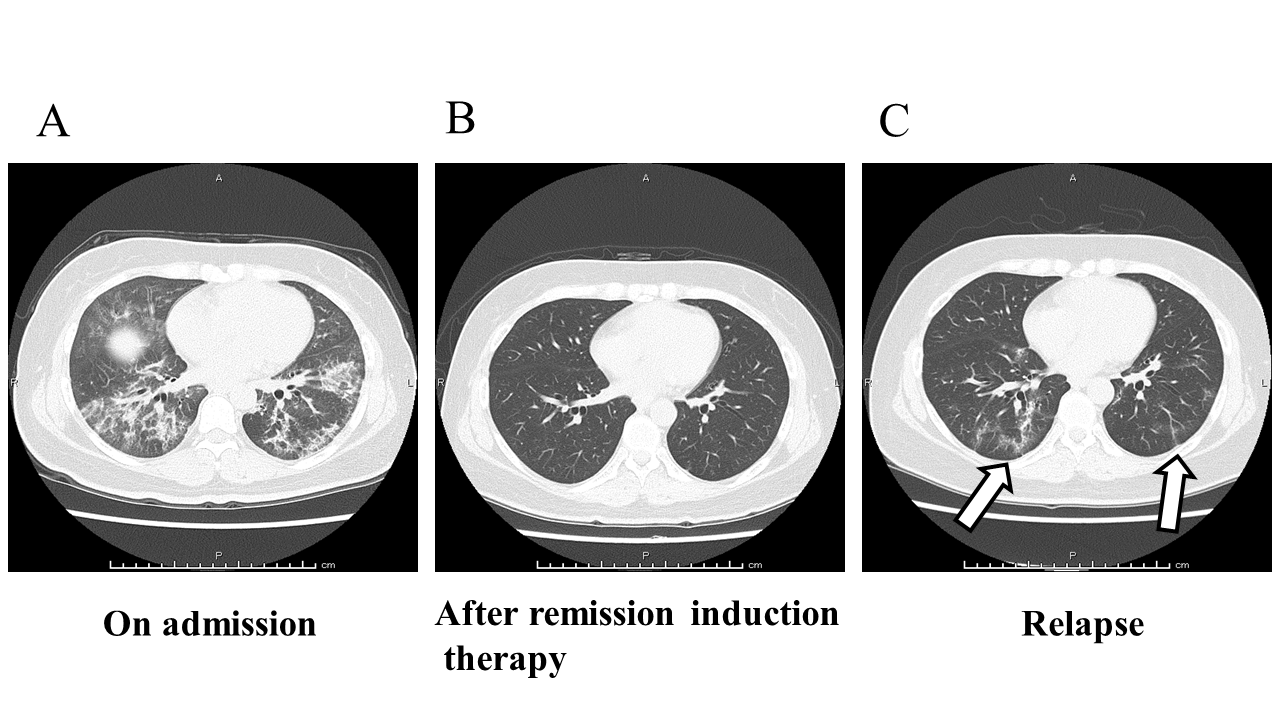

Supplement: Supplementary Figure 1 — The representative images of relapse in patients with anti-ARS-ILD. (A), chest HRCT image on admission. (B), chest HRCT image after remission induction therapy. (C), chest HRCT image on relapse. On chest HRCT, GGO was seen in bilateral lower lobes (wide arrow). ARS, Aminoacyl-tRNA Synthetase; ILD, interstitial lung disease; HRCT, high-resolution computed tomography; GGO, Ground-glass opacity. [file Image1.tif]

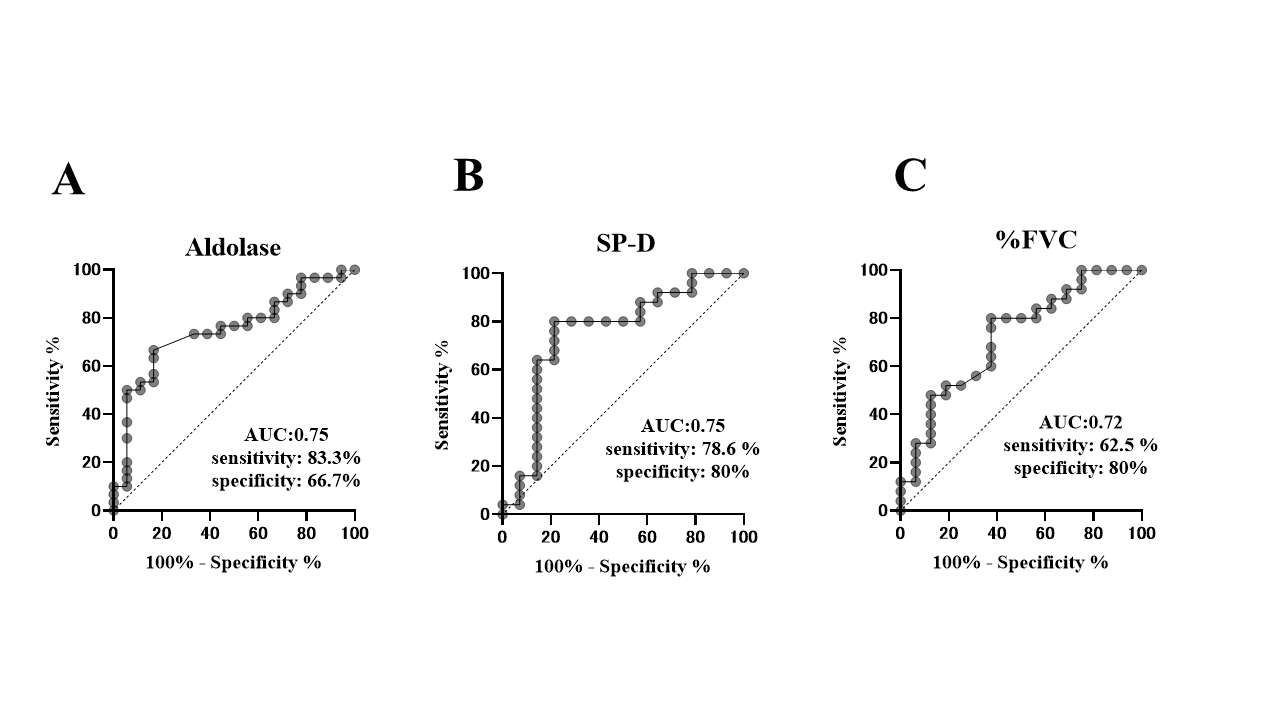

Supplement: Supplementary Figure 2 — The ROC curves of aldolase (A), SP-D (B), %FVC (C) for differentiating patients with relapse from patients without relapse in anti-ARS-ILD. ROC, receiver operating characteristic; SP-D, surfactant protein-D; FVC, forced vital capacity; ARS, Aminoacyl-tRNA Synthetase; ILD, interstitial lung disease; AUC, area under the curve. [file Image2.tif]
