# Supplementary material for: Poor prognostic factors for relapse of interstitial lung disease with anti-aminoacyl-tRNA synthetase antibodies after combination therapy
Source: Front Immunol. 2024 Sep 13;15:1407633. doi: 10.3389/fimmu.2024.1407633 (PMC11427292; doi:10.3389/fimmu.2024.1407633)
Supplement: Supplementary file 3 [file Table1.docx]

| **Supplementary Table 1. Comparison of contents of treatment between relapsed group and non-relapsed group** | | | |
| --- | --- | --- | --- |
| **Characteristics** | **Relapsed group (n= 19)** | **Non-Relapsed group (n=31)** | ***P*** |
| PDN (n=50), mg/day | 55 (45-60) | 45 (35-50) | 0.02^*^ |
| CSA (n=14), mg/day | 275（244-356）^a^ | 250（206-294）^b^ | 0.27 |
| TAC (n=36), mg/day | 6（4.5-9）^c^ | 6（4-8）^d^ | 0.21 |
| MPDN pulse, n (%) | 3（15.8） | 7（22.6） | 0.72 |
| IVCY, n (%) | 12（63.2） | 12（38.7） | 0.15 |
| IVIG, n (%) | 1（5.3） | 0（0） | 0.38 |
| PE, n (%) | 0(0) | 2(6.5) | 0.52 |
| MMF, n (%) | 1(5.3) | 1(3.2) | 1.00 |
| The laboratory markers are　presented as the median (interquartile range). The P-values were estimated using Fisher's exact test or Wilcoxon rank sum test. **P* <0.05. PDN: prednisolone; CSA: cyclosporine; TAC: tacrolimus; MPDN: methylprednisolone; IVCY: intravenous pulse cyclophosphamide; IVIG: intravenous immunoglobulin; PE: plasma exchange; MMF: mycophenolate mofetil. ^a^Number of subjects, n= 6. ^b^Number of subjects, n= 8. ^c^Number of subjects, n= 13. ^d^Number of subjects, n= 23. | | | |
